# Iron and Alzheimer’s Disease: From Pathology to Imaging

**DOI:** 10.3389/fnhum.2022.838692

**Published:** 2022-07-13

**Authors:** Dean Tran, Phillip DiGiacomo, Donald E. Born, Marios Georgiadis, Michael Zeineh

**Affiliations:** ^1^Department of Radiology, Stanford School of Medicine, Stanford, CA, United States; ^2^Department of Pathology, Stanford School of Medicine, Stanford, CA, United States

**Keywords:** Alzheimer’s disease, iron imaging, iron imaging methods, iron pathology, iron MRI, iron microscopy methods, iron X-ray methods, iron mechanism

## Abstract

Alzheimer’s disease (AD) is a debilitating brain disorder that afflicts millions worldwide with no effective treatment. Currently, AD progression has primarily been characterized by abnormal accumulations of β-amyloid within plaques and phosphorylated tau within neurofibrillary tangles, giving rise to neurodegeneration due to synaptic and neuronal loss. While β-amyloid and tau deposition are required for clinical diagnosis of AD, presence of such abnormalities does not tell the complete story, and the actual mechanisms behind neurodegeneration in AD progression are still not well understood. Support for abnormal iron accumulation playing a role in AD pathogenesis includes its presence in the early stages of the disease, its interactions with β-amyloid and tau, and the important role it plays in AD related inflammation. In this review, we present the existing evidence of pathological iron accumulation in the human AD brain, as well as discuss the imaging tools and peripheral measures available to characterize iron accumulation and dysregulation in AD, which may help in developing iron-based biomarkers or therapeutic targets for the disease.

## Introduction

Alzheimer’s disease (AD) is a debilitating illness which afflicts millions worldwide, but no effective treatment exists. This is partly due to the lack of understanding of the molecular basis behind the disease.

Presently, AD pathology is typically characterized by an abnormal accumulation of β-amyloid in plaques and phosphorylated tau in neurofibrillary tangles leading to neurodegeneration including synaptic and neuronal loss. Support for a role of β-amyloid comes from genetic risk factors—mutations in amyloid precursor protein (APP) and presenilin genes lead to β-amyloid accumulation and confer risk to early-onset AD (Goate et al., 1972; Levy-Lahad et al., 1995; Sherrington et al., 1995; Tanzi, 2012). However, despite this accumulation, β-amyloid plaques correlate poorly with memory decline (Giannakopoulos et al., 2003; Ingelsson et al., 2004). Interestingly, this correlation does improve for soluble amyloid (Tomic et al., 2009). In contrast, while a genetic linkage is not established between genes associated with processing tau and AD (Goedert, 2005), its accumulation in neurofibrillary tangles has a strong correlation with neurodegeneration and memory decline (Duyckaerts et al., 1998; Giannakopoulos et al., 2003; Ingelsson et al., 2004). Diagnosis of AD requires the presence of both β-amyloid and tau accumulations, although the mechanisms by which they produce neurodegeneration remain unknown (van der Kant et al., 2020), and attempts to treat AD by targeting β-amyloid and tau pathology have been unsuccessful in showing conclusive cognitive improvement (Kametani and Hasegawa, 2018). Even FDA-approved amyloid clearance that is thought to address amyloid (including soluble amyloid) does not have a clear clinical benefit (Knopman et al., 2020). Thus, β-amyloid and tau do not seem to provide a complete explanation of the pathogenesis and progression of AD.

Recent work suggests that microglial inflammation also plays an important role in AD, and may link β-amyloid and tau pathology with neurodegeneration. Genetic analyses demonstrated increased AD risk associated with inflammation-related genes including TREM2 (Guerreiro et al., 2013; Jonsson et al., 2013), CD33 (Bradshaw et al., 2013; Griciuc et al., 2013), and microglia genes (Sims et al., 2017). Gene expression studies show AD associated remodeling of microglial gene regulatory networks (Zhang et al., 2013) and increased hippocampal major histocompatibility complex (MHC) II expression localized to AD microglia, which was inversely correlated with cognitive ability (Parachikova et al., 2007). Furthermore, evidence shows microglia interact extensively with β-amyloid and tau. *In vivo* preclinical models demonstrate that microglia phagocytize injected fibrillary amyloid (Geula et al., 1998; Weldon et al., 1998), β-amyloid plaques increase microglial reactivity, and soluble amyloid oligomers can activate microglia (Yang et al., 2017). Depletion of murine microglia can also reduce the trans-synaptic spread of tau (Asai et al., 2015). Additionally, the importance of inflammation is suggested by cellular responses—pro-inflammatory cytokines are increased in AD (Morimoto et al., 2011).

Iron is a key component of inflammation; thus, it is of direct importance to AD. Additionally, it is important in many other fundamental biological processes in the brain including oxygen transportation, DNA synthesis, mitochondrial respiration, myelin synthesis, and neurotransmitter synthesis and metabolism (Ward et al., 2014). As early as 1953, abnormal iron accumulation in the hippocampus has been associated with AD (Goodman, 1953). Since then, much work has been done examining the role of iron and iron homeostasis in relation to AD pathology. In this review, we will present the existing evidence and mechanistic insights of iron involvement in AD, and discuss novel imaging tools and peripheral measures available to characterize iron accumulation and dysregulation in AD.

## Biological Evidence of Iron Alterations in the Alzheimer’s Disease Brain

### Iron Cycle in the Brain

**FIGURE 1:**
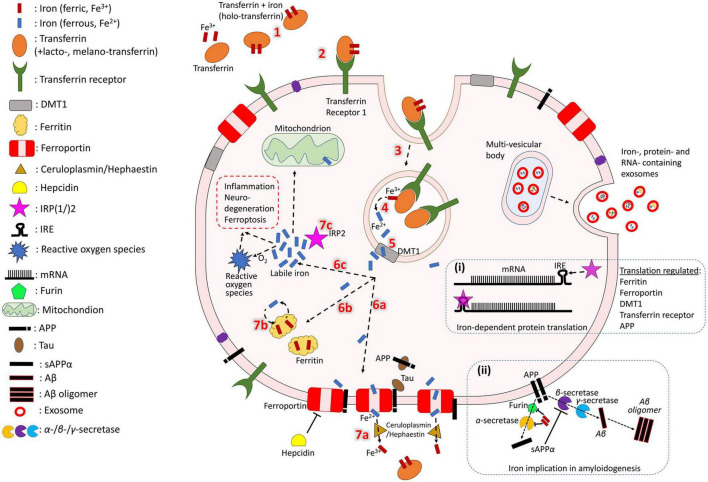
Iron cycle in brain cells. Transferrin carrying Fe^3+^ (1) binds to transferrin receptor (2) and is endocytosed (3). Fe^3+^ is reduced to Fe^2+^ (4) and exported to the cytosol by DMT-1 (5). In the cell, iron may have different fates. It can be exported by ferroportin (6a), which is stabilized by APP (trafficked with the help of tau) and downregulated by hepcidin. Fe^2+^ is oxidized by ceruloplasmin as it re-binds to transferrin (7a). Iron can also be stored in ferritin as Fe^3+^ (6b) and released when needed (7b). Fe^2+^ in the cell can act as labile iron (6c), which is used by cellular processes and generates reactive oxygen species for cell metabolism (which could also trigger inflammation and cytotoxicity). The extent of the labile iron pool is monitored by iron regulatory proteins (IRP) (7c). Iron, iron-related proteins and RNAs can also be exported from the cell through exosomes (right side). Box (i) shows mRNA transcription of iron-associated proteins which is regulated by IRPs and miRNAs. Box (ii) shows iron regulating activity of furin, which cleaves and activates alpha-secretase, whose sAPP production inhibits beta-secretase-driven amyloidogenesis.

Iron normally enters the brain by crossing the blood-brain barrier *via* transferrin receptor-1 mediated endocytosis of transferrin (an iron transport protein) or non-bound iron (Moos and Morgan, 2000; Ke and Qian, 2007; Moos et al., 2007; Leitner and Connor, 2011). In AD, however, it can also enter without endocytosis when there is breakdown of the blood-brain-barrier (BBB) (van de Haar et al., 2016) or recruitment of iron-containing peripheral macrophages and resident microglia by β-amyloid plaques followed by apoptosis (Hohsfield and Humpel, 2015). Local iron stores are then recycled throughout the brain *via* export and transport mechanisms which support the diverse functions of various cell types (neurons, microglia, astrocytes, oligodendrocytes, etc.). While these mechanisms have not been fully elucidated, an approximate model of iron cellular physiology in brain cells is depicted in Figure 1. Transferrins carrying ferric iron (Fe^3+^) bind to transferrin receptor-1 and are endocytosed. Fe^3+^ is then reduced to ferrous iron (Fe^2+^) and exported to the cytosol *via* divalent metal transporter 1 (DMT1). In the cell, iron may be exported by ferroportin (an iron export protein stabilized by APP and downregulated by hepcidin) and Fe^2+^ can be oxidized by ceruloplasmin, a major antioxidant protein, as it re-binds to transferrin. Iron can also be stored in ferritin (iron storage protein) as Fe^3+^ and released when needed. Additionally, labile iron (free Fe^2+^ in the cytosol) can be used by cellular processes, is modulated by iron regulatory proteins (IRP), or generate reactive oxygen species for cell metabolism.

### Histological Iron Staining

**FIGURE 2:**
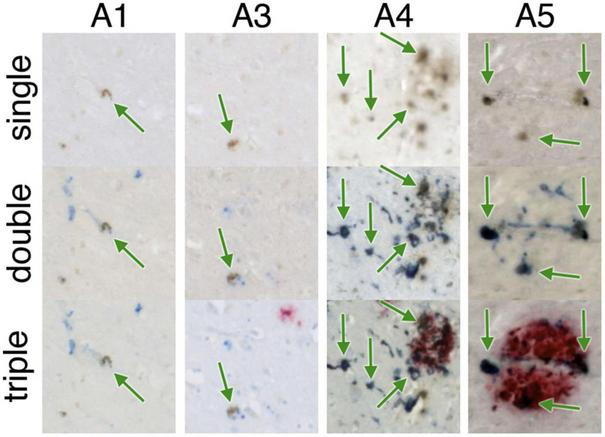
Iron-containing microglia in the subiculum. A1-A5 represent different AD specimens. Single = 3,3′-diaminobenzidine (DAB)-iron (brown), double = DAB-iron (brown) plus CD163, a marker for microglia (blue), triple = DAB-iron (brown) plus CD163 (blue) plus β-amyloid (red). On all of these hippocampal slides, iron-containing microglia are evident as brown spots surrounded by blue stain, sometimes but not always associated with β-amyloid plaques. Figure from Zeineh et al. (2015).

**FIGURE 3:**
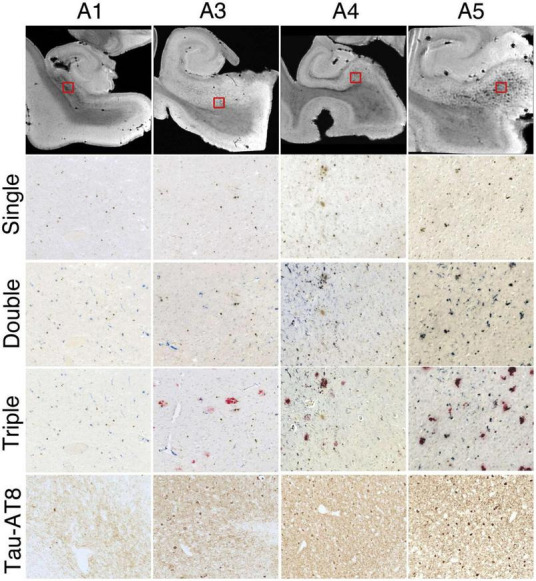
Successive histologic images demonstrating consistent iron and microglia at the subiculum for 4 hippocampal specimens. Top row: representative magnetic resonance imaging virtual slices from each specimen. Successive rows: zoomed in histological sections. Single = 3,3′-diaminobenzidine (DAB)-iron (brown), double = DAB-iron (brown) plus CD163-microglia (blue), triple = DAB-iron (brown) plus CD163-microglia (blue) plus β-amyloid (red). Brightness, contrast, and color balance were adjusted homogeneously across each image to evenly emphasize iron deposits, microglia, β-amyloid deposits, and neurofibrillary tangles as well as neuropil threads. Figure from Zeineh et al. (2015).

Multiple validated histological techniques exist for iron as well as iron related proteins. Perls’ Prussian blue iron stain is the best-known histological technique used to visualize the distribution of iron in AD specimens. Specifically, potassium ferrocyanide, K_4_[Fe(CN)_6_], reacts with Fe^3+^ ions to give an insoluble, intense Prussian blue pigment, as well as with Fe^2+^, resulting in a white precipitate called Everitt’s salt that eventually oxidizes to give a similar blue hue. Furthermore, treatment of tissue with acid can ionize iron bound to iron metabolism proteins, such as transferrin, hemosiderin and ferritin, allowing visualization of these species, while it cannot highlight iron present in hemoglobin or neuromelanin due to strong covalent bonding and affinities (Meguro et al., 2007). 3,3′-Diaminobenzidine (DAB) can be used to intensify Perls’ staining—examples can be seen in Figure 2 (top row) and Figure 3 (single). The Turnbull technique is also a common related histochemical method. This technique consists of applying potassium ferricyanide, K_3_[Fe(CN)_6_], in association with hydrochloric acid, making it possible to reveal Fe^2+^ ions. Variations of the Turnbull stain using pretreatments can also allow visualization of Fe^3+^ ions (de Barros et al., 2019).

Ferrous (Fe^2+^, redox-active) iron accumulation is associated with senile plaques and neurofibrillary tangles in human AD (Smith et al., 1997) and murine APP models of AD (Falangola et al., 2005). DAB-enhanced Perls’ iron staining has shown iron accumulation in the AD hippocampus, as shown in Figure 2 (Zeineh et al., 2015).

### Proposed Mechanisms of Iron in Alzheimer’s Disease

#### Amyloid and Tau

Mechanistically, excess intracellular iron prevents iron regulatory proteins (IRP) from binding to iron-response elements (IREs) in the 5′ untranslated region of APP mRNA (Zhou and Tan, 2017), resulting in increased APP, which could predispose to increased β-amyloid AD pathology. Iron can also inhibit alpha-secretase-induced APP cleavage *via* furin, leading to excess beta-secretase cleavage of APP and increased β-amyloid production (Silvestri and Camaschella, 2008). Additionally, DMT-1 is colocalized with β-amyloid in plaques of the AD brain, suggesting its involvement in APP processing and β-amyloid generation (Zheng et al., 2009).

Iron is also linked to tau (Rao and Adlard, 2018), promoting tau phosphorylation (Wan et al., 2019) and inducing hyperphosphorylated tau aggregation into neurofibrillary tangles (Yamamoto et al., 2002). High dose iron treatment on APP mice increased tau phosphorylation and treatment of APP mice with deferoxamine, an iron chelator, abolished tau phosphorylation (Guo et al., 2013), reduced β-amyloid deposition, inhibited apoptosis in the brain, and improved cognitive function (Zhang and He, 2017).

It is important to note, however, that there are distinct contrasts between human AD brain tissue and the APP mouse model—iron distribution, iron management, and glial response histologically differ between the two (Meadowcroft et al., 2015).

#### Inflammation

Iron may play a role in microglial driven inflammation, an important process in AD pathology (Geula et al., 1998; Weldon et al., 1998; Parachikova et al., 2007; Bradshaw et al., 2013; Griciuc et al., 2013; Guerreiro et al., 2013; Jonsson et al., 2013; Asai et al., 2015; Sims et al., 2017; Yang et al., 2017). In fact, microglia in the human AD hippocampus contains increased concentrations of iron storage protein ferritin (Grundke-lqbal et al., 1990; Connor et al., 1992a; Meadowcroft et al., 2015). This accumulation of iron in microglia occurs by increasing expression of iron import protein DMT-1 and simultaneously decreasing expression of iron export protein ferroportin (Rathore et al., 2012; Urrutia et al., 2013). Redox-active iron accumulations are associated with glial cells (Smith et al., 2010), and DAB-enhanced Perls’ iron staining has shown iron within activated microglia in the subiculum of the hippocampus, as shown in Figure 2 (Zeineh et al., 2015). Similarly, β-amyloid plaque infiltrating microglia in the neighboring middle temporal gyrus show increased ferritin expression (Kenkhuis et al., 2021). This microglial accumulation of iron in inflammation is confirmed by *in vivo* animal models (Urrutia et al., 2013; McIntosh et al., 2019). Treatment of microglia with proinflammatory cytokines causes an increase in iron uptake, while treatment with anti-inflammatory cytokines causes iron retention, further suggesting the association of iron with microglial inflammation (Rathore et al., 2012).

Toxic levels of ferrous (Fe^2+^) iron in human AD tissue can also promote inflammation by serving as a catalyst for the production of free radicals *via* the Fenton reaction (Smith et al., 1997; Quintana et al., 2004; Pankhurst et al., 2008). A cellular stress protein (coded by the *heme oxygenase-1* gene) that oxidizes heme to biliverdin, iron, and carbon monoxide in response to noxious stimuli, is significantly overexpressed in neurons and astrocytes of the AD hippocampus and cerebral cortex, which could produce iron in the AD brain (Schipper et al., 1995). Glial heme oxygenase-1 expression in the temporal cortex and hippocampus is significantly greater in AD (Schipper et al., 2006). Additionally, ceruloplasmin, a major plasma antioxidant that converts redox-active iron back to Fe^3+^, is increased in AD, suggesting a responsive increase to oxidative stress (Loeffler et al., 1996).

#### Cell Death and Ferroptosis

Iron may be linked to cell death mechanisms in AD (Perry et al., 2002; Hambright et al., 2017; Masaldan et al., 2019). One such mechanism is iron-dependent ferroptosis (Dixon et al., 2012) which involves lipid peroxidation, an event that occurs early in AD progression (Praticò and Sung, 2004), and may be contributing to AD neurodegeneration (Jakaria et al., 2021). Evidence for ferroptotic mechanisms is found in post-mortem AD tissue (Ashraf et al., 2020b), as well as in AD mouse models (Bao et al., 2021). Delaying ferroptosis in mice produces reductions in β-amyloid (Raefsky et al., 2018), neuronal death and memory impairment (Bao et al., 2021), while destruction of ferroptosis inhibiting proteins such as GPX4 accelerates cognitive decline (Hambright et al., 2017). Indeed, depletion of these ferroptosis-inhibiting proteins (such as glutathione) in the frontal cortex and hippocampus has been observed in AD (Mandal et al., 2015). Ferroptosis mechanism and the evidence in AD were also recently reviewed elsewhere (Ashraf and So, 2020; Lane et al., 2021; Zhang et al., 2021).

#### Dysregulation of Iron Homeostasis

Supporting the hypothesis that iron is linked to AD pathology is the abundant evidence of several dysregulated iron-related proteins in AD (Table 1), including alterations in levels of iron-transport proteins that could exacerbate iron’s interactions with amyloid and tau. Overall activity of transferrin, an iron binding protein responsible for delivery and transport of iron to cells in serum, lymphatic fluid, and cerebrospinal fluid (CSF), is decreased in AD. Concentrations of transferrin are consistently lower in white matter of various cerebral cortical regions (Connor et al., 1992b), and transferrin receptor densities and transferrin binding are significantly reduced in the AD hippocampus (Kalaria et al., 1992; Morris et al., 1994). On the other hand, lactotransferrin, a transferrin glycoprotein, and iron scavenger (Kell et al., 2020), is labeled *via* immunohistochemistry within neurons and glia in human AD brains (Kawamata et al., 1993). Moreover, lactotransferrin colocalizes with neurofibrillary tangles in the hippocampal formation and inferior temporal cortex and correlates with antibody stains of β-amyloid and tau (Leveugle et al., 1994). Additionally, transcriptomics on a very large cohort showed lactotransferrin correlated with β-amyloid burden, while *in vitro* data point to its involvement in amyloidogenesis (Tsatsanis et al., 2021). Similarly, melanotransferrin (p97) is found in elevated levels associated with microglial cells in senile plaques (Yamada et al., 1999). Ferritin, the major iron storage protein, seems to be increased in activated microglia in the AD temporal gyrus (Kenkhuis et al., 2021). Levels of ferroportin, the main iron exporter protein that requires APP for stability in neurons (Wong et al., 2014), are significantly decreased in the AD hippocampus (Raha et al., 2013), and single nucleotide polymorphisms in *SLC 40A1*, the gene coding ferroportin, are significantly associated with AD (Crespo et al., 2014). Also, ferroportin is downregulated in mouse AD models, and its reduced levels are associated with hippocampal atrophy, memory deficits and ferroptotic cell phenotype, while restored levels seem to prevent ferroptosis and memory impairment (Bao et al., 2021). Similarly, hepcidin, a peptide hormone that seems to modulate the action of ferroportin, has been found to be decreased in AD hippocampi (Raha et al., 2013), while hepcidin’s overexpression in mouse astrocytes protects against β-amyloid-induced neurodegeneration (Zhang et al., 2020).

**TABLE 1:** Alterations in measures of iron-related proteins.

Protein	Role	Alteration (analyzed biofluid)	References
Transferrin (+ lacto-,melano-transferrin)	Transport	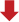 (Serum) 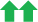 (Serum; saliva)	Fischer et al., 1997; Kwan Kim et al., 2001; Carro et al., 2017
Transferrin receptor	Import	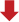 (Brain tissue)	Morris et al., 1994
Ferritin	Storage	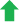 (CSF)	Ayton et al., 2015
Ferroportin	Export	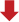 (Brain tissue)	Raha et al., 2013
DMT-1	Import/export	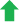 (Brain tissue)	Zheng et al., 2009
Ceruloplasmin	Oxidation	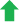 (Serum)	Squitti et al., 2011
Hepcidin	Regulation	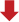 (Brain tissue)	Raha et al., 2013

#### Evidence From Risk Factors Linking Iron and Alzheimer’s Disease

Iron overload disorders have linked iron to AD and neurodegeneration. The iron overload disorder hereditary hemochromatosis itself is strongly associated with progression of dementia in both males and females (Percy et al., 2014). Patients who underwent blood transfusions, which often results in iron overload and is linked with dysregulated iron metabolism (Perrotta and Snyder, 2001), and significantly higher risk of dementia and AD (Lin et al., 2019). The apolipoprotein E gene is significantly associated with AD (Crespo et al., 2014), while a *H63D* mutation in this gene appears protective against AD (Percy et al., 2008).

#### Integrative Mechanism

**FIGURE 4:**
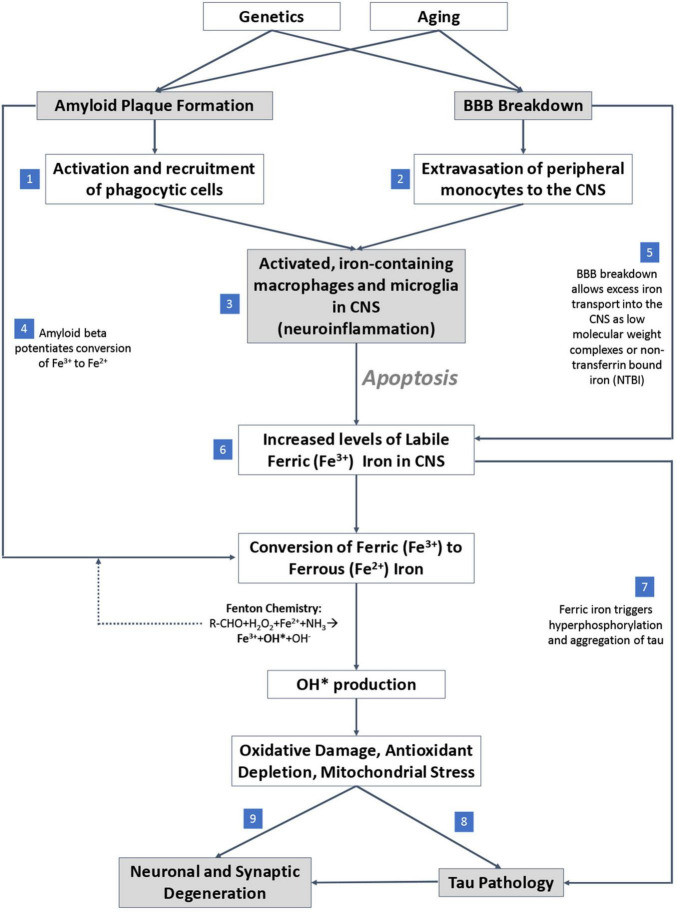
Proposed mechanistic pathway of iron mediated neurodegeneration. (1) β-amyloid plaques potentiate activation and recruitment of phagocytic cells in AD. (2) Blood brain barrier (BBB) breakdown leads to extravasation of peripheral monocytes to CNS in AD. (3) Increase in activated inflammatory cells in CNS in AD. (4) β-amyloid potentiates conversion of ferric to ferrous iron. (5) BBB breakdown allows free iron directly into CNS. (6) Accumulation of labile iron in CNS (increase in iron levels) in AD. (7) Ferric iron triggers tau phosphorylation and aggregation. (8) Oxidative damage drives tau pathology. (9) Excess iron and associated oxidative stress induce ferroptosis, a direct cell death mechanism.

An overview of the mechanisms by which iron accumulation occurs in the AD brain and drives neurodegeneration is summarized in Figure 4. First, iron accumulation in the brain can occur through breakdown of the blood-brain-barrier (BBB) (van de Haar et al., 2016), which could allow for diffusion of free iron into the brain from the periphery. β-amyloid plaques recruit and activate iron containing peripheral macrophages and resident microglia, leading to neuron apoptosis (Hohsfield and Humpel, 2015) which, in combination with BBB breakdown, can potentially drive extravasation of peripheral iron containing monocytes to the CNS. The reactive monocytes increase their iron content *via* DMT-1 and downregulation of iron-export pathways (Recalcati et al., 2012) and ultimately undergo apoptosis, releasing labile intracellular iron into surrounding tissue (Reif and Simmons, 1990; Wirenfeldt et al., 2007; Neher et al., 2011; Rathnasamy et al., 2013). The labile iron directly affects oxidative stress and free radical production in AD by way of β-amyloid protofibrils, which interact with ferric (Fe^3+^) iron to produce ferrous (Fe^2+^) iron, a catalyst for toxic free radicals *via* Fenton chemistry (Huang et al., 1999; Everett et al., 2014). Overall, the accumulated iron produces reactive oxygen species that can damage DNA (Melis et al., 2013), alter DNA expression by epigenetic mechanisms (Kwok, 2010), induce post translational modifications to proteins (Perluigi et al., 2012), and drive tau pathology (Mondragón-Rodríguez et al., 2014), suggesting iron’s importance as a potential early driver in AD.

### Iron Involvement in Non-Alzheimer’s Disease Neurodegenerative Pathology

Iron is also implicated in other neurodegenerative disorders, including Parkinson’s disease (PD), Huntington’s disease (HD), amyotrophic lateral sclerosis (ALS), and neurodegeneration with brain iron accumulation (NBIA). Its role in these diseases appears in some ways to overlap with proposed mechanisms driving neurodegeneration in AD, and in other ways appears secondary.

Iron accumulation is present in the neurons and glia of the substantia nigra in patients with Parkinson’s disease—in fact, iron concentrations correlate with disease severity (Dexter et al., 1987; Dexter et al., 1989; Hirsch et al., 1991; Pyatigorskaya et al., 2020). As in AD, accumulation of iron in PD has been linked to increased BBB permeability (Kortekaas et al., 2005) and microglial-mediated neuroinflammation (Conde and Streit, 2006). In PD, studies have shown an increase of lactoferrin receptors in neurons (Faucheux et al., 1995), increased expression of DMT1 in dopamine neurons (Salazar et al., 2008), disruption of transferrin receptor type 2 (Mastroberardino et al., 2009), and mutations in various genes relevant to iron transport, including the hemochromatosis gene (Borie et al., 2002; Guerreiro et al., 2006).

Iron accumulation is identified in the striata and basal ganglia of patients with Huntington’s disease (Bartzokis et al., 1999, 2007; Jurgens et al., 2010), predominantly within reactive astrocytes (Bulk et al., 2020b), suggestive of a link to neuroinflammation (Crotti and Glass, 2015). However, it seems to be secondary—*huntingtin* (the gene mutated in Huntington’s disease) is involved in iron homeostasis regulation (Hilditch-Maguire et al., 2000), and mutated *huntingtin* results in increased expression of iron response proteins, inducing iron overload (Chen et al., 2013; Niu et al., 2018).

In amyotrophic lateral sclerosis (ALS), there is increased iron accumulation by microglia around the motor cortex (Kwan et al., 2012). The mechanisms underlying this finding are unclear, though abnormal iron homeostasis inducing excessive oxidative stress in motor neurons has been postulated to contribute to disease pathogenesis (Carrì et al., 2003).

Neurodegeneration with brain iron accumulation (NBIA) is a group of genetic disorders characterized by the abnormal accumulation of iron in the basal ganglia (Gregory and Hayflick, 2013). There are many variations caused by genetic mutations of proteins involved in various pathways, including iron homeostasis, coenzyme A biosynthesis, lipid metabolism, and autophagy, among others, though the specific role the mutations play in inducing iron accumulation remain unclear (Levi and Finazzi, 2014; Levi and Tiranti, 2019). Further study of these disorders may provide insights into the mechanisms linking iron accumulation to neurodegeneration.

### Colocalization of Other Elements With Iron in Alzheimer’s Disease

Several other transition metals have been studied in relation to oxidative stress in the context of AD. Overall, high levels of zinc, iron, and copper are found in amyloid plaques and neurofibrillary tangles of AD (Lovell et al., 1998; Roberts et al., 2012; James et al., 2016). In particular, copper, alongside iron, has been shown to induce aggregation of β-amyloid (Lovell et al., 1998) and contribute to neuronal oxidative stress (Sayre et al., 2000). Overall copper levels are decreased in the AD brain (Schrag et al., 2011; Kozlowski et al., 2012).

The role of Zn in β-amyloid aggregation is also unclear (Wojtunik-Kulesza et al., 2019). Zinc deficiency has been shown to accelerate AD-like memory deficits in brains of APP mice without modifying β-amyloid plaque burden (Rivers-Auty et al., 2021). The association of copper and zinc with plaques is more dependent on metal concentrations in surrounding neuropil than iron, which has a lower affinity to the β-amyloid peptide (James et al., 2016).

MRI and autopsy studies have also found deposits of gadolinium, a paramagnetic heavy metal used for contrast enhancement in MR imaging, in sites of iron accumulation in the brain, particularly in the iron-rich dentate nucleus and globus pallidus. Gadolinium arrives exclusively because of intravenous administration, and small amount of unchelated gadolinium is presumably absorbed. Interestingly, different absorption rates are found depending on the exact compound used (Radbruch et al., 2015). Nevertheless, the possible colocalization of these two elements in the brain may motivate research into overlapping mechanisms of accumulation (Ramos et al., 2014; Acosta-Cabronero et al., 2016; El-Khatib et al., 2019; Mallio et al., 2020).

## *Ex vivo* Imaging/Measurement

*Ex vivo* imaging techniques leveraging specific properties of iron, stemming from its atomic structure or the magnetic properties of its subatomic particles, have been used to visualize abnormal iron accumulation in the brain and elucidate chemical speciation and redox state. On their own, these methods are not functional measures of iron—thus, they are typically coupled with other methods of iron analysis or histology, which can provide the necessary biological context. For an overview of the different methods, their working mechanism and resolution (see Box 1).

**Box 1. Iron imaging/measurement methods.**

**METHODS BASED ON ATOMIC STRUCTURE**

**X-ray methods**

**X-ray fluorescence (XRF)**

**FIGURE 5:**
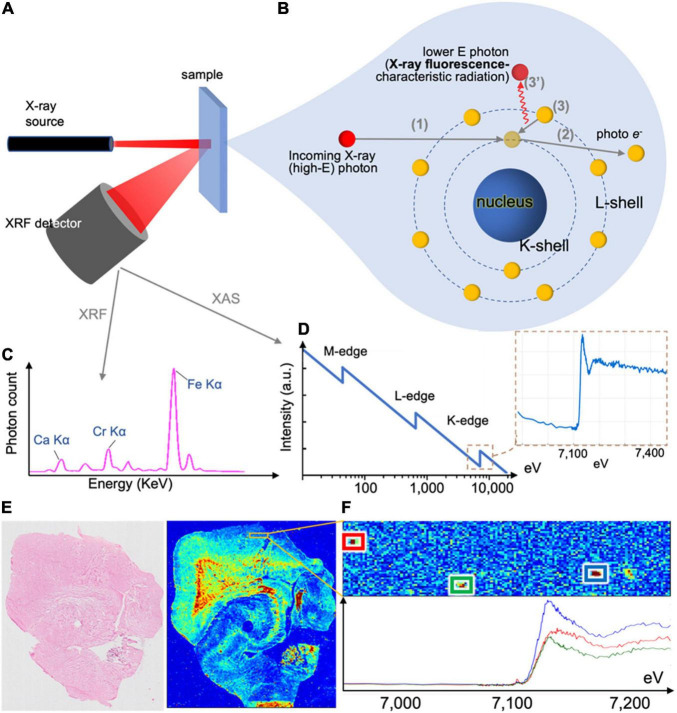
X-ray methods’ working principle and example outputs. **(A)** X-ray fluorescence (XRF) setup, with incoming beam, sample and photon spectrometer collecting emitted photons of all energies. The same setup can be used in X-ray absorptions spectrometry (XAS) or X-ray absorption near-edge structure (XANES), where the incoming beam energy is modulated and output recorded. **(B)** Incoming photons interact with the sample, and displace inner shell electrons, which are emitted as photoelectrons. When these are replaced by outer shell electrons, the change of energy of the latter leads to the emission of a photon with energy corresponding to the energy difference of the two shells, which is characteristic of the element. Emitted photons are collected by the photon spectrometer. **(C)** XRF spectrum collected by the photon spectrometer in **(A)**. The photon count in each energy bin can provide elemental concentrations in the sample. **(D)** Typical XAS and XANES spectra corresponding to iron. **(E)** When the beam raster-scans the tissue section (left) and an XRF spectrum is collected for each point, the output can be an elemental map of the sample (here an iron map of a human hippocampus). **(F)** XANES spectra can be collected for multiple identified iron deposits.

In XRF (Zhang et al., 2018), a photon beam of fixed energy impinges on the sample and displaces electrons from the sample atoms. Displaced inner shell electrons are replaced by outer shell electrons, with concomitant emission of photons (Figures 5A,B). The energy of the emitted photons is specific to the element (Figure 5C), corresponding to the energy difference of the atom shells (i.e., characteristic radiation). Emitted photons are collected by the X-ray fluorescence detector, and the acquired spectrum reveals the sample’s elemental content. Raster-scanning the sample enables generation of elemental (iron) maps (Figure 5E), and can measure multiple elements but cannot directly measure changes related to oxidation state. Resolution depends on the beam diameter, and can reach nanometer-sizes (Chevrier et al., 2022).

**X-ray Absorption Near Edge Structure (XANES)**

In XANES (Alp et al., 1990), subset of X-ray Absorption Spectroscopy (XAS), the setup can be similar to XRF (alternatively, it is in transmission mode). The incoming beam energy is modulated around the absorption edge of an element of interest (Figure 5D). The resulting spectrum can provide extra information such as the oxidation state of iron deposits (Figure 5F). Resolution depends on the beam size, and, similar to XRF, can reach nanometer range (Chevrier et al., 2022).

**Proton(/particle)-induced X-ray emission (PIXE)**

PIXE (Verma, 2007) uses a similar concept to XRF (Figures 5A,B), but instead of high-energy photons, the incoming beam that displaces inner-shell electrons consists of protons, or other charged particles/ions. Micro particle-induced X-ray emission (micro-PIXE) analysis can provide information on the elemental distribution in tissue with good spatial resolution (on the order of 0.5-50 μm) and excellent multielement capability, including K, Ca, Mn, Fe, Cu, and Zn (Maenhaut, 2019), but not oxidation state.

**Scanning Transmission X-ray Microscopy (STXM)**

In STXM (Takeichi, 2018), a monochromatic X-ray beam of fixed energy (in the soft X-ray range of a few hundred eVs) is focused on the sample, and transmitted photons are recorded by an X-ray detector downstream. The sample is raster-scanned, and a high-resolution transmission image for this energy is created. This is performed for multiple incoming beam energies, and in the end a spectrum for each sample point is generated, and the energy range spans the absorption edge of both organic (e.g., C, N) and inorganic elements (e.g., Fe and Ca), without the use of staining. Spatial resolution can reach several nanometers (Lermyte et al., 2019). Magnetically sensitive STXM X-ray magnetic circular dichroism (XMCD) further allows for detection of alterations related to oxidation state (Everett et al., 2018).

For all X-ray methods, the sample is typically in the form of thin sections (since fluorescence photons cannot travel very far in tissue) and is raster-scanned by the X-ray beam to create elemental or oxidation state maps.

**Ion methods**

**Inductively Coupled Plasma (ICP) Mass Spectrometry (MS)/Optical(/Atomic) Emission Spectrometry (OES/AES)**

ICP spectrometry methods use plasma to ionize the sample, and break it into smaller, charged particles. In ICP-MS (Wilschefski and Baxter, 2019) these particles are then accelerated, deflected, and detected based on their mass-to-charge ratio, leading to identification of the ions and elements in the sample, with excellent sensitivity. Coupling ICP-MS to methods such as liquid chromatography or capillary electrophoresis can enable element speciation analysis in liquid samples (Michalke, 2002; Michalke et al., 2019). Alternatively, in ICP-AES (Olesik, 1991) phenomena similar to those in the X-ray methods are exploited, whereby excited atoms return to their ground state and emit photons, which reflect the element-specific energy differences between shells. Use of laser ablation (LA) enables raster-scanning the sample, ablating each point before the subsequent ICP-MS/AES analysis (LA-ICP-MS/AES), to create elemental maps of iron (but not oxidation state) with lateral resolution at micrometers range.

**Secondary Ion Mass Spectroscopy (SIMS)**

SIMS (Nuñez et al., 2018) is similar in concept to LA-ICP-MS/AES, but instead of the laser ablation and ICP, the sample is raster-scanned with a primary ion beam, which directly ablates and ionizes the sample locally. Charged particles are then separated based on the charge-to-mass ratio similar to ICP-MS. Scanning with the ion beam allows for nanometer-resolution elemental imaging (NanoSIMS) with the ability to detect iron, though not directly discriminate oxidation state (Kilburn and Wacey, 2015).

**Neutron methods**

**Instrumental Neutron Activation Analysis (INAA)**

In INAA (Malainey, 2011) the sample is irradiated with neutrons, generating radioactive isotopes of its elements. The emitted gamma radiation is collected and matched to the known element emission spectra, facilitating elemental analysis of the elements that form radionuclides, such as trace metals. Samples can be very small (in the milligrams range) but also large, and do not typically require any sample preparation, since neutrons and gamma rays are highly penetrating.

**Electron methods**

**Energy Dispersive (X-ray) Spectroscopy (EDS/EDX)**

The principle of EDS (Scimeca et al., 2018) is similar to XRF, but instead of an X-ray beam, inner-shell electrons are displaced by the electron beam in the scanning or transmission electron microscope. An X-ray spectrometer, similar to the XRF setup, is needed to analyze the lower energy photons, in order to identify elements in the sample by their characteristic radiation. Elemental maps can be then created (including Fe, Ca, and Si), with spatial resolution ranging from 10 nm to a few micrometers, though EDS is not able to distinguish iron oxidation states.

**Electron Energy Loss Spectroscopy (EELS)**

EELS is conceptually similar to XAS, enabling analysis of elements like Fe, but it can also discern properties such as oxidation state. In a transmission electron microscope (TEM), the electrons impinging on the sample can scatter inelastically, losing energy when interacting with the sample atoms. An electron spectrometer collects transmitted electrons, with subsequent evaluation of how much energy has been lost compared to the original beam energy. The loss spectrum has peaks at values corresponding to the energy required for displacing inner shell electrons of the sample atoms. Resolution is in the nanometer range (Hofer et al., 2016).

**METHODS BASED ON MAGNETIC PROPERTIES**

**Superconducting Quantum Interference Device (SQUID) magnetometry**

SQUID magnetometry (Buchner et al., 2018) is a highly sensitive method that can measure very weak magnetic fields down to 10^–14^ T. The method utilizes the paired electrons of two superconductors in one or two Josephson junctions, and can detect magnetic fields as well as magnetic moments, measuring susceptibility. There are different modes of measurement, such as the magnetization vs. field (M-H) curves, magnetization vs. temperature (M-T) curves, or the isothermal remanent magnetization (IRM) curves, with the latter being most commonly used for measuring iron in tissues. Combination of the modes can be used to distinguish iron oxidation states and quantify magnetite or ferrihydrite in tissue. Samples are usually in powder/pellet form.

**Electron Paramagnetic Resonance (EPR) or Electron Spin Resonance (ESR) spectroscopy**

EPR (Calas, 2018) exploits the fact that unpaired sample electrons are aligned parallel or antiparallel to an externally applied magnetic field, similar to proton alignment in MRI. The resonance frequency is determined by an externally applied field (typically at the microwave range), while the magnetic field is swept until the energy provided to the system by the field matches the energy of the microwaves for the given quantity of free electrons. The method can analyze iron content of minute pieces of tissue (including oxidation state), down to the micrometer scale.

**Magnetic Resonance Imaging (MRI)**

MRI is a tomographic method for imaging larger samples than the other methods. In MRI, signal depends on the relaxation properties of magnetic spins in the sample, most commonly hydrogen protons, found most abundantly in water molecules. In the presence of elements such as iron, the relaxation properties of the water protons are altered, most notably the T2* relaxation, which depends on local field inhomogeneities. Susceptibility-weighted imaging generates contrast based on phase images and serves as a more qualitative way to display magnetic field variations in tissue (Liu et al., 2015). A more quantitative way to potentially image elements such as iron in tissue is to estimate the local susceptibility (quantitative susceptibility mapping, QSM) (Ruetten et al., 2019), since the presence of iron in the tissue changes the local tissue susceptibility. By complex mathematical algorithms, QSM reconstructs distributions of magnetic dipoles (representing the local susceptibility of tissue) to match the magnetic field inhomogeneities obtained from the phase image of gradient-echo images. QSM has typical MRI resolutions in the mm range *in vivo*, to the hundreds or even tens of micrometers range in *ex vivo* specimens. The sensitivity of MRI imaging increases at higher magnetic field strengths (Deistung et al., 2013). Unlike other imaging methods mentioned, MRI serves as indirect imaging of iron and thus cannot differentiate between molecular forms of iron and cannot provide information about elemental specificity.

### X-ray Microscopy

The use of X-ray microscopy techniques to visualize iron accumulation in the *ex vivo* brain is well established. In particular, X-ray fluorescence (XRF) (Figures 5A–C,E) is frequently used to measure total brain iron in AD (Hopp et al., 2010; Zheng et al., 2012). X-ray Absorption Near Edge Structure (XANES) (Figures 5D,F) can determine the location and specific chemical composition of iron deposition. XANES has been used to quantify concentrations of ferritin and magnetite (a magnetic iron oxide) in the AD frontal lobe (Collingwood et al., 2005), as well as to show higher iron accumulation in neuritic plaques vs. in non-neuritic plaques, with ferrous iron (Fe^2+^) more abundant in the former and ferric (Fe^3+^) in the latter (Álvarez-Marimon et al., 2021). Micro-PIXE demonstrates a significant increase in iron in the rim and core of neuritic plaques in the AD amygdala (Lovell et al., 1998). X-ray absorption spectroscopy and transmission X-ray microscopy (which can visualize the structure and composition of β-amyloid aggregates) have been combined with X-ray magnetic circular dichroism spectroscopy (which observes the magnetic properties of metals) and iron assay quantification to show that accumulation of β-amyloid can mediate reduction of Fe^3+^ to redox-active Fe^2+^, serving as a direct source of oxidative stress in AD (Huang et al., 1999; Everett et al., 2014). This has been replicated using XANES and electron microscopy in Fe^3+^ ferritin-bound iron being converted to ferrous state in the presence of β-amyloid (Everett et al., 2020), further pointing toward a role of β-amyloid in oxidative stress.

### Ion Microscopy

Ion microscopy techniques such as inductively coupled plasma mass spectrometry (ICP-MS) and secondary ion mass spectroscopy (SIMS) have been used to study iron in the AD brain. ICP-MS can detect metals in samples at very low concentrations, including iron. ICP-MS analyses show iron concentration elevation in AD brains correlating with Braak stages (classifications of the degree of pathology in AD) (Szabo et al., 2016), higher iron levels in AD hippocampi than controls (Cruz-Alonso et al., 2019), and altered iron distribution (but no significant difference in iron levels) in the cortex of AD patient vs. controls (Bulk et al., 2020a). Higher iron (and zinc) content was also found using ICP-MS in the brains of transgenic tau mice (Solovyev et al., 2021). SIMS allows for direct identification of chemical elements with high sensitivity and specificity. In the context of AD, it permits the visualization of iron distribution on AD hippocampal tissue—NanoSIMS analysis in AD has shown the presence of iron at the periphery of senile plaques and in some glial cells (Quintana et al., 2006).

### Neutron Analysis

Neutron Activation Analysis can be used to identify trace metals in *ex vivo* specimens, with high specificity. It has shown increased iron concentrations in cortical specimens from AD brains (Andrási et al., 1995), the olfactory pathways (Samudralwar et al., 1995), as well as in the hippocampus (Deibel et al., 1996). Moreover, increased iron in the inferior temporal cortex assessed post-mortem was strongly associated with accelerated cognitive decline in the final 12 years of life (Ayton et al., 2020).

### Electron Microscopy

**FIGURE 6:**
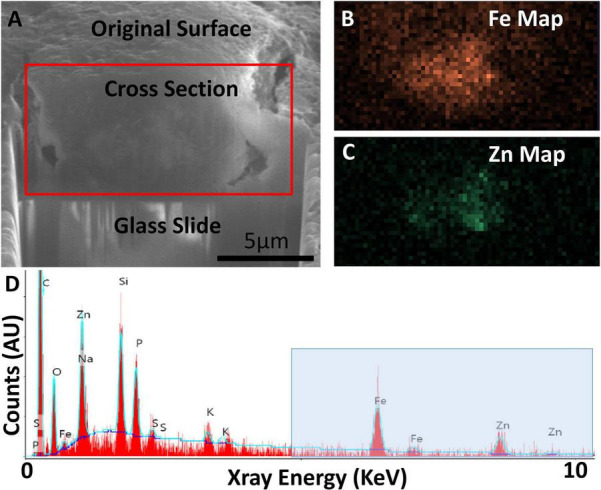
Focused ion beam scanning electron microscopy (FIB-SEM) exposing cross-section of an AD specimen **(A)** with SEM-EDS confirming presence of iron (Fe) and zinc (Zn) **(B–D)**. **(A)** Secondary electron SEM (SE-SEM) image of the exposed cross section, with the glass slide/substrate visible below. The scale bar is the same for **(A–C)**, and is accurate only in the indicated direction as the sample is viewed at an angle. **(B)** EDS iron map at 6.4 keV acquired on the boxed region from **(A)**, confirming the presence of an iron-rich feature. **(C)** EDS zinc map at 8.6 keV acquired on the boxed region from **(A)**, confirming the partially overlapping presence of a zinc-rich feature. **(D)** Overview EDS spectrum integrated across the tissue cross section, showing iron as well as zinc. Peaks for common organic elements [carbon (c), phosphorus (P), sodium (Na), sulfur (S)] are present, as well as silicon (Si) and oxygen (O) from the glass. The blue box indicates the energy space relevant for identifying iron and zinc. The blue line depicts a background fit. Figure from Madsen et al. (2020).

**FIGURE 7:**
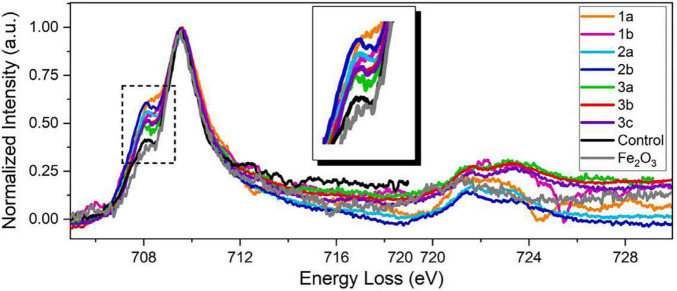
STEM-EELS dataset acquired from three different AD brain tissue samples. An EEL spectra overlay of AD specimens, a negative control specimen, and a Fe_2_O_3_ standard specimen (99.9%, Sigma-Aldrich). All spectra from AD brain tissues exhibit stronger pre-peaks than those from the negative control specimen and the Fe_2_O_3_ standard, suggesting higher concentration of ferrous ions in AD brain specimens. The pre-peak intensity of the negative control specimen is at the same level as the Fe_2_O_3_ standard, suggesting that iron deposits in healthy brains are mainly comprised of ferric iron. The different pre-peak intensities in AD brain tissues also suggest variations of ferrous/ferric iron concentrations of different AD patients and deposits. Figure from Zheng et al. (2021).

Electron microscopy can provide quantifications of iron with nanometer resolution in AD. Analytical transmission electron microscopy (ATEM) has been used to identify iron related proteins such as ferritin and hemosiderin at the ultrastructural level by combining traditional electron microscopy with high resolution X-ray nanoanalysis to verify presence of iron in molecular cores. Specifically, both ferritin and hemosiderin are observed in the periphery of senile plaques and glial cells and coincide with NanoSIMS analyses of iron (Quintana et al., 2006). Other electron microscopy techniques, namely scanning electron microscopy (SEM), energy dispersive spectroscopy (EDS), and electron energy loss spectroscopy (EELS) have also shown an increase of redox active iron (Fe^2+^) not present in controls, shown in Figures 6, 7 (Madsen et al., 2020; Zheng et al., 2021 supporting the notion that iron is a key player in AD oxidative stress. Electron nanodiffraction, high resolution transmission electron microscopy, and electron energy loss spectroscopy (EELS), which are commonly used in nanoanalysis of materials, can also been used to analyze structure of individual ferritin protein cores in AD (Quintana et al., 2004). These have shown dysfunction of ferritin with an increase in toxic brain ferrous irons contributing to the production of free radicals, inducing both oxidative stress and myelin breakdown associated with cognitive decline in AD (Quintana et al., 2006).

### Magnetometry

Magnetic properties of AD brain tissue can be examined using superconducting quantum interference device (SQUID) magnetometry, a highly sensitive method of precisely measuring extremely small magnetic field changes. Due to its high sensitivity, this method is appropriate for measuring weakly magnetic materials such as magnetite, a magnetic iron oxide which has been shown to accumulate in AD and produce free-radicals *via* the Fenton reaction. Still, estimating concentrations of magnetite is difficult due to interference from diamagnetic signals of tissue, and also ferritin, which has significantly different magnetic properties—as a result, complementary magnetic measurements are often performed to separate magnetite signals from background (Hautot et al., 2003). When used to examine AD brain samples, SQUID magnetometry showed a significantly higher concentration of magnetite in AD, implicating iron accumulation in AD oxidative stress (Hautot et al., 2003; Pankhurst et al., 2008). Of note, the iron accumulation was on average greater in women. Increased ferrihydrite, an idrited form of iron, was also observed in severe AD patients compared to controls, although magnetite levels did not differ (van der Weerd et al., 2020). On the other hand, combination of SQUID with EPR showed higher ferrihydrite concentration and higher magnetite magnetic moment in AD brains (Bulk et al., 2018).

### Magnetic Resonance Imaging

High field magnetic resonance imaging (MRI) is sensitive to microscopic iron and can be used to visualize iron deposits in the *ex vivo* human/critter brain at resolutions ∼100 μm, with the possibility of translation to *in vivo imaging*. Increases in the presence of ferric iron in brain tissue cause faster T2* relaxation in *ex vivo* 7 Tesla MRI, so regions of high iron content are clearly visible on MR images with varying degrees of signal dropout based on the amount of iron present at that location. T2*-weighted 7T gradient echo MR images of *ex vivo* tissues show a loss of signal that coregisters with histological iron and β-amyloid stains, with iron deposits colocalized around β-amyloid (Meadowcroft et al., 2009). Similarly, QSM was used to show that β-amyloid-rich frontal gray matter had higher susceptibility values, suggestive of higher iron content, compared to plaque-free gray matter (Tiepolt et al., 2018). QSM at 9.4T and 14.1T has also been used to identify β-amyloid-rich cortical areas, results which extended to *in vivo* MR (Tuzzi et al., 2020), presented later. Similar results but with tau tangles have been shown in mouse models of AD (O’Callaghan et al., 2017). Along the same lines, R2* (R2* = 1/T2*) distributions in the AD hippocampus correspond with increased iron deposition (Antharam et al., 2012). Moreover, 7T MR using a modified balanced steady-state free precession sequence (bSSFP), which allows for a high contrast resolution, alongside a gradient echo (GRE) sequence, both sensitive to iron, coregistered with histologic staining and microscopy found MRI hypointensities in the subiculum corresponding to microscopic iron in activated microglia, shown in Figure 7 (Zeineh et al., 2015). This is in support of the inflammatory component to iron’s involvement in AD as described above (see section “Proposed Mechanisms of Iron in Alzheimer’s Disease”).

## *In vivo* Imaging and Biomarkers

*In vivo* imaging and peripheral measures have been used to quantify iron and iron-related proteins, bridging the gap between central and peripheral processes and suggesting potential for translation to non-invasive biomarkers.

### *In vivo* Magnetic Resonance Imaging

MRI has been extensively used to map iron in AD (Langkammer et al., 2014). Initial efforts with MRI utilized spin-echo T2 relaxation principles (iron deposition shortens T2 relaxation times) to detect possible iron alteration in AD. In particular, 3T MRI imaging had shortened T2 relaxation times in the AD hippocampus, correlating to increases in iron deposition (Schenck et al., 2006). Similarly, AD mice (5xFAD) on an iron-rich diet showed shortened T2 in somatosensory cortex, while T2 values showed an interplay with inflammation (Aljuhani et al., 2020). Still, T2 shortening is not iron-specific– other mechanisms including changes in myelin density can contribute to this phenomenon (Schenck et al., 2006). Field dependent R2 increase (FDRI) MRI, which can quantify tissue iron by calculating the difference in R2 (inverse of T2) on two different field strengths, found significantly higher iron quantities in AD patients in the caudate and globus pallidus compared to controls (Bartzokis et al., 1994), as well as changes in the hippocampus (Raven et al., 2013). However, FDRI is not a specific measure of iron, and the mechanism producing it is not clearly understood.

**FIGURE 8:**
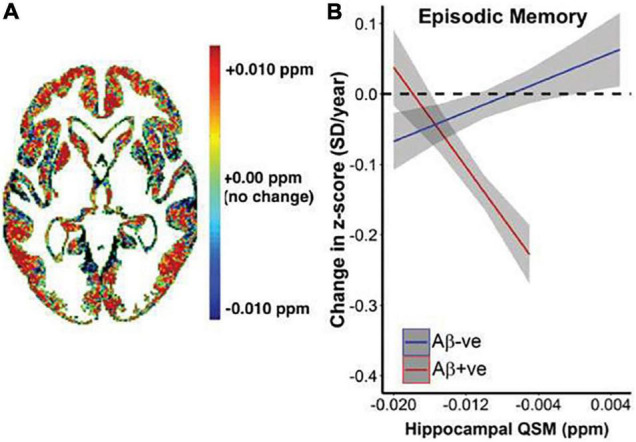
Relationship between QSM, β-amyloid, and longitudinal cognitive outcomes. **(A)** Heat map of average difference in QSM between β-amyloid positive subjects β-amyloid negative subjects. **(B)** Graphical representation of the impact of baseline QSM upon change of cognition, measured by cognitive composite scores of episodic memory (California Verbal Learning Test-delayed, Logical Memory Test-delayed, Rey Complex Figure Test-Long Delay). Annual rate of cognitive decline was calculated for each individual based on his or her trajectory of decline for the entire period they were included in the study. This is plotted against baseline QSM value. Baseline QSM in the hippocampus was negatively associated with annual rate of change in episodic memory in β-amyloid positive subjects (*P* = 9.2*10^– 7^), and positively associated with rate of change in β-amyloid negative subjects (*P* = 0.014). Figure from Ayton et al. (2017b).

Susceptibility imaging using gradient echo sequences can measure brain iron accumulation *in vivo*, by quantifying signal and phase changes caused by iron deposition, which is more accurate, sensitive, consistent, and efficient than T2 (Haacke et al., 2007; Ding et al., 2009). Phase imaging applied to AD patients found lower phase values in the AD basal ganglia and hippocampus, suggesting increased iron concentrations (Ding et al., 2009). Quantitative susceptibility mapping (QSM) can also provide estimation of iron *in vivo*, by deconvoluting unwrapped phase images and providing a distribution of magnetic susceptibility in the tissue (Ravanfar et al., 2021), which is more selective for iron than T2* relaxometry (Ayton et al., 2017b). QSM used alongside β-amyloid positron emission tomography (PET) found that hippocampal iron levels predicted accelerated cognitive deterioration in individuals with β-amyloid pathology (Figure 8; Ayton et al., 2017b). A comprehensive review of applications of QSM in AD and other neurodegenerative diseases can be found in Ravanfar et al. (2021).

### Biofluid Measures of Iron

While MRI can potentially demonstrate the levels and distribution of iron in AD *in vivo*, peripheral measures of iron or iron-related proteins (see Table 1) can provide more specific measurements of dysregulated iron transporters and metabolites. Several studies have measured iron related proteins in cerebrospinal fluid (CSF), serum, and saliva in relation to AD progression and cognitive decline.

Lower transferrin serum levels are correlated with lower cognitive function scores (Fischer et al., 1997), lower hippocampal volume and higher amyloid deposition -assessed by PET (Ashraf et al., 2020a). Moreover, genetic analysis of blood shows significant associations with AD for single nucleotide polymorphisms in transferrin (*TF*) and transferrin receptor (*TFR2*) genes (Crespo et al., 2014). Similarly, lower CSF melanotransferrin (p97) levels correlate with a reduction in hippocampal volume over time (Ashraf et al., 2019a). Patients with dementia caused by AD also differ in serum melanotransferrin (p97) levels—melanotransferrin levels were elevated three to fourfold in AD compared to non-AD dementia and normal controls (Kwan Kim et al., 2001). Salivary lactoferrin (also known as lactotransferrin), an antimicrobial peptide that can regulate inflammatory response by decreasing iron overload and reactive oxygen formation, shows promise as a biomarker for AD. In fact, accuracy for AD diagnosis by salivary lactotransferrin levels was greater than diagnosis by CSF biomarkers, including phosphorylated tau (Carro et al., 2017). Higher CSF levels of ferritin were found to be associated with worse cognitive performance, and accelerated pathology and cognitive decline (Ayton et al., 2015, 2017a,^2018^). Ferritin is also a promising serum marker: increased serum levels correlate with lower scores of tests of cognitive function (i.e., higher dementia levels) (Fischer et al., 1997). Multi-element analysis of AD serum using XRF showed lower iron levels in AD vs. controls (Ashraf et al., 2019b). A serum iron status profile analysis showed a decrease of iron, ferritin, transferrin, ferroportin, and transferrin receptor concentrations in AD patients (Crespo et al., 2014). Corresponding with lower ferroportin levels were higher observed levels of hepcidin in AD patient serum compared to controls (Sternberg et al., 2017), although the study did not test for a correlation with cognitive decline. While the fluid biomarker studies above generally showed decreased levels of iron-related proteins, a study examining ceruloplasmin to transferrin ratio in serum found that transferrin saturation, and not transferrin itself, actually increased in AD patients (Squitti et al., 2011).

## Discussion and Future Directions

The literature supports iron’s involvement in AD—evidence of iron alterations has been consistently observed *ex vivo*, with histology, biological techniques, and imaging, and *in vivo*, with MR and biofluid measures. Despite the evidence presented here that iron accumulation and dysregulation is a hallmark feature in the AD brain, it remains an outstanding question whether it is simply the result of other underlying pathologic drivers (which may make it a good biomarker of disease, but not a therapeutically relevant target) or if it has a causative role in driving neurodegeneration (with potential for targeted therapies).

There exists limited evidence supporting iron as a causative factor in AD. Proteomic studies show that binding of ferrous iron to β-amyloid peptides facilitates aggregation through formation of β-sheet structures (Boopathi and Kolandaivel, 2016). *In vitro* work has also suggested a causative role for iron in the formation of β-amyloid aggregates—treatment of neurons with ferric citrate facilitated non-amyloidogenic processing of APP (Chen et al., 2018). Similarly, treatment of β-amyloid peptides with ferric citrate promotes aggregation (Galante et al., 2018) and iron chelation decreases it (Tahmasebinia and Emadi, 2017). *In vivo*, increasing the brain iron of APP mice through dietary overload has been shown to potentiate iron accumulation in amyloid plaques and microgliosis (Peters et al., 2016). It is important to note, however, that there are distinct contrasts between human AD brain tissue and the APP mouse model—iron distribution, iron management, and glial response histologically differ between the two (Meadowcroft et al., 2015). However, further research with preclinical AD models and clinical trials of iron modulation in humans are needed to potentially provide evidence for a mechanistic link between iron and Alzheimer’s pathology and progression.

Advances in MRI to increase accuracy and specificity of iron detection may possibly reveal early AD pathology. Initial work has demonstrated the potential of *in vivo* MRI techniques to detect iron deposition and dysregulation in AD. Leveraging ultra-high-field (7T) MRI systems, which can produce images with higher signal-to-noise ratio (SNR) and spatial resolution than lower-field strength MR systems (1.5T, 3T), may provide additional research and clinical benefit. These systems are being used to elucidate novel insights into the pathology of neurological and neuropsychiatric diseases *ex vivo* (de Reuck et al., 2014; Yao et al., 2014; Zucca et al., 2016; Kenkhuis et al., 2019). These findings have led to a growing number of *in vivo* studies performed at 7T, where increased resolution of structural and functional scans has led to enhanced visualization of microstructures (Deistung et al., 2013; Bianciardi et al., 2018) and novel networks of brain connectivity (Muckli et al., 2015), enabling insights into the pathology of a range of neurological diseases (de Graaf et al., 2012; van Veluw et al., 2013; Harrison et al., 2015; de Ciantis et al., 2016; Obusez et al., 2018; Shah et al., 2019). In AD, high-resolution 7T brain imaging, when compared to 3T, has made several advancements in our understanding of disease pathology and pathogenesis and may potentially improve timely diagnosis and management (Yedavalli et al., 2021). 7T imaging can be used alongside β-amyloid PET-MR and tau PET-MR for a full analysis of AD pathology *in vivo*. However, high SNR and resolution requires long scan times resulting in image artifacts caused by patient motion, which limits the clinical interpretability of images and the reliability of quantitative analyses, such as segmentations that estimate volume and thickness. Thus, work is being done to accelerate scans (Uðurbil et al., 2019) or implement motion correction systems (DiGiacomo et al., 2020).

Additionally, while evidence of iron dysregulation has been seen with peripheral measures, the current data have not translated to strong diagnostic or prognostic markers. Extracellular vesicles such as exosomes, which have been linked to AD spread and progression in preclinical models (Gallart-Palau et al., 2020) and AD-derived cells (Ruan et al., 2021), have also been shown to transport iron (Truman-Rosentsvit et al., 2018) and increase its export in order to lower oxidative stress and inhibit ferroptosis (Brown et al., 2019). Hence, they are being increasingly studied as AD biomarkers (Malm et al., 2016; Quek and Hill, 2017; Lee et al., 2019; Hornung et al., 2020). Iron related proteins found in exosomes include: transferrin and its receptor (Harding et al., 1983; Pan and Johnstone, 1983), ferritin (Truman-Rosentsvit et al., 2018), ceruloplasmin (Gudehithlu et al., 2019), lactoferrin (Wu and Liu, 2018), melanotransferrin (Greening et al., 2016), DMT-1 (Mackenzie et al., 2016), hepcidin (Sasaki et al., 2019), and ferroportin (Chawla et al., 2019). Extracellular vesicles may thus play a role in iron or iron-related protein and RNA (inter-cellular) transport, in neurodegeneration and specifically in AD (Zhang et al., 2021), though their importance in iron-related pathways in AD remains to be investigated.

Further investigation of iron in AD can elucidate the mechanisms behind AD pathogenesis and progression, while also allowing potential for translation to clinical applications using iron as an AD biomarker or even therapeutic target.

## Author Contributions

MZ, PD, and MG conceived the manuscript idea. DT wrote the manuscript with the help of all authors. All authors planned the manuscript and contributed to revision of the manuscript.

## Conflict of Interest

The authors declare that the research was conducted in the absence of any commercial or financial relationships that could be construed as a potential conflict of interest.

## Publisher’s Note

All claims expressed in this article are solely those of the authors and do not necessarily represent those of their affiliated organizations, or those of the publisher, the editors and the reviewers. Any product that may be evaluated in this article, or claim that may be made by its manufacturer, is not guaranteed or endorsed by the publisher.
